# *Viburnum stellato-tomentosum* Extract Suppresses Obesity and Hyperglycemia through Regulation of Lipid Metabolism in High-Fat Diet-Fed Mice

**DOI:** 10.3390/molecules26041052

**Published:** 2021-02-17

**Authors:** Seona Cho, Hwa Lee, Jisu Han, Haneul Lee, Rosales Ovares Kattia, Zamora Villalobos Nelson, Sangho Choi, Soo-Yong Kim, Ho-Yong Park, Hye Gwang Jeong, Tae-Sook Jeong

**Affiliations:** 1 Industrial Bio-Materials Research Center, Korea Research Institute of Bioscience and Biotechnology (KRIBB), Daejeon 34141, Korea; csa0916@kribb.re.kr (S.C.); leehua@kribb.re.kr (H.L.); jisu.han@kipox.re.kr (J.H.); lhn713@kribb.re.kr (H.L.); hypark@kribb.re.kr (H.-Y.P.); 2 College of Pharmacy, Chungnam National University, Daejeon 34134, Korea; 3 Instituto Nacional de Biodiversidad (INBio), Santo Domingo, Heredia, P.O. Box 22-3100, Costa Rica; krosales@inbio.ac.cr (R.O.K.); askinbio@inbio.ac.cr (Z.V.N.); 4 International Biological Material Research Center, KRIBB, Daejeon 34141, Korea; decoy0@kribb.re.kr (S.C.); soodole@kribb.re.kr (S.-Y.K.)

**Keywords:** *Viburnum stellato-tomentosum*, amentoflavone, obesity, hyperglycemia, lipogenesis, β-oxidation, insulin sensitivity

## Abstract

The potential biological activities of *Viburnum stellato-tomentosum* (VS), a plant mainly found in Costa Rica, have yet to be reported. Supplementation of VS extract for 17 weeks significantly decreased body weight gain, fat weight, fasting glucose, insulin, homeostasis model assessment of insulin resistance (HOMA-IR), and triglyceride levels in high-fat diet (HFD)-fed C57BL/6J mice. The molecular mechanisms underlying the anti-obesity and glucose-lowering effects of VS extract were investigated. VS extract suppressed adipocyte hypertrophy by regulating lipogenesis-related CCAAT/enhancer-binding protein α (C/EBPα) and insulin sensitivity-related peroxisome proliferator-activated receptor γ (*Pparg*) expression in adipose tissue (AT) and hepatic steatosis by inhibiting C/EBPα and lipid transport-related fatty acid binding protein 4 (FABP4) expression. VS extract enhanced muscular fatty acid β-oxidation-related AMP-activated protein kinase (AMPK) and PPARα expression with increasing *Pparg* levels. Furthermore, VS extract contained a much higher content of amentoflavone (AMF) (29.4 mg/g extract) compared to that in other *Viburnum* species. AMF administration decreased *Cebpa* and *Fabp4* levels in the AT and liver, as well as improved insulin signaling-related insulin receptor substrate 1 (*Irs1*) and glucose transporter 1 (*Glut1*) levels in the muscle of HFD-fed mice. This study elucidated the *in vivo* molecular mechanisms of AMF for the first time. Therefore, VS extract effectively diminished obesity and hyperglycemia by suppressing C/EBPα-mediated lipogenesis in the AT and liver, enhancing PPARα-mediated fatty acid β-oxidation in muscle, and PPARγ-mediated insulin sensitivity in AT and muscle.

## 1. Introduction

Obesity is a state of excessive body fat caused by a chronic imbalance between energy intake and energy expenditure, which results in an increased risk of related comorbidities, including insulin resistance and type 2 diabetes mellitus (T2DM) [1]. High-fat diets (HFD), such as Western diets, are characterized by overnutrition, leading to weight gain and obesity due to the presence of excess body fat. The global predominance of people who are overweight and obese has increased rapidly since the 1980s and is now considered a major risk factor in the 21st century, as well as an epidemic that threatens the well-being of the global population [2]. Insulin resistance (IR), caused by obesity and T2DM, results in poor insulin function in glucose uptake, metabolism, and storage. In addition, these functional defects may impair insulin signaling in insulin-sensitive tissues, including the skeletal muscle, liver, and adipose tissue [3].

White adipose tissue (WAT), where energy is stored in the form of lipids, is strongly associated with obesity. Increased fat mass, particularly hypertrophy and hyperplasia, elevates plasma free fatty acids. Increased release of fatty acids from the expanded fat tissue can impair hepatic metabolism and induce hepatic steatosis with lipid accumulation, which is often accompanied by glucose intolerance [3,4,5]. Increases in the presence of hepatic steatosis are related to adverse changes in glucose, fatty acid, and lipid metabolism, with rising trends in obesity [6]. Moreover, adiponectin, an adipocyte-derived hormone and an insulin-sensitive effector, is highly expressed in WAT. The expression of adiponectin is downregulated in obesity, while several factors, such as leptin, tumor necrosis factor α (TNFα), interleukin (IL)-6, and resistin, are overproduced [7]. Furthermore, IR causes defects in the β-oxidation of long-chain fatty acids, resulting in altered glucose and lipid metabolic pathways in the skeletal muscle [8]. As a result, a significant accumulation of unused triglycerides (TG) is negatively associated with insulin sensitivity [9].

The genus *Viburnum* is the most familiar representative of the family *Caprifoliaceae* in the American tropics. *Viburnum stellato-tomentosum* (VS), a cultivable protected plant, is a semi-rare shrub-like tree that is mainly distributed in the deep mountains of Costa Rica and Panama. It is a round-topped tree with black edible fruit known as “tirra,” “curra,” or “sura” [10]. Previous studies have reported that amentoflavone (AMF) is a special biflavone biosynthesized in the genus *Viburnum*, with a slightly higher concentration in the leaves than in the branchlets [11]. A previous study reported that intraperitoneal injection of AMF exerts body weight-lowering effects and protection against HFD-induced metabolic dysfunction by reducing fasting glucose, insulin, homeostasis model assessment of insulin resistance (HOMA-IR), and TG levels in HFD-fed rats. AMF also influenced adipogenesis in differentiated 3T3-L1 adipocytes, leading to a reduction in the gene and protein expression of peroxisome proliferator-activated receptor γ (PPARγ), CCAAT/enhancer-binding protein α (C/EBPα), and C/EBPβ [12]. However, to the best of our knowledge, nothing is known about the active compounds and beneficial bioactivities of VS. In this study, we investigated the effects and in vivo molecular mechanism of VS extract and its major compound, AMF, on obesity and hyperglycemia in HFD-fed C57BL/6J mice for the first time. To elucidate the molecular mechanism underlying the beneficial regulation by VS extract in dietary obese mice, we assessed the reductions in body and fat weight gain, glucose homeostasis, and the expression of genes and proteins related to lipid metabolism and fatty acid β-oxidation, including fatty acid transport and adipocyte differentiation, in the WAT, liver, and skeletal muscle. Moreover, the expression of insulin signaling-related genes was also examined.

## 2. Results

### 2.1. HPLC Analysis of VS Extract

**Figure 1:**
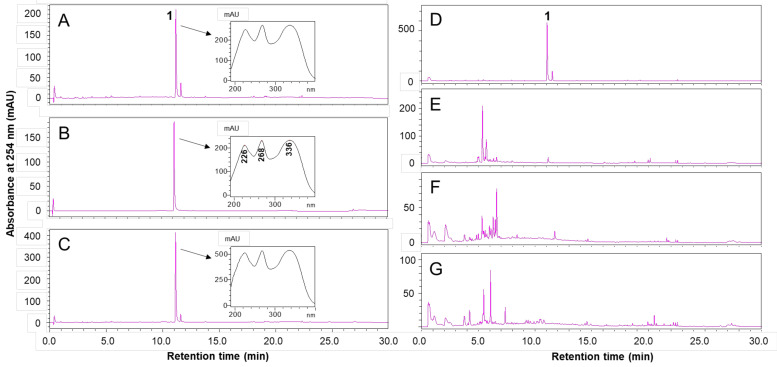
High-performance liquid chromatography (HPLC) profiles of 95% ethanol extracts from the aerial parts of *Viburnum stellato-tomentosum* (VS) and the other *Viburnum* species were detected at 254 nm. HPLC chromatograms of (**A**,**D**) VS extract (A: 5 mg/mL and D: 10 mg/mL), (**B**) amentoflavone (AMF, 125 μg/mL), (**C**) co-injection with VS extract (5 mg/mL) and AMF (125 μg/mL), (**E**) *V. lantana* L. extract (10 mg/mL), (**F**) *V. opullus* var. sargentii (Koehne) Takeda extract (10 mg/mL), and (**G**) *V. dillatatum* Thunb. extract (10 mg/mL).

High-performance liquid chromatography (HPLC) profiles of the VS extract, AMF, and co-injection with VS extract and AMF detected by HPLC-diode array detection (HPLC-DAD) at 254 nm are shown in Figure 1A–C. The retention times and UV spectra of compound **1** in the VS extract and AMF were the same as those in the HPLC profiles. Compound **1** was isolated from the ethyl acetate fraction of the 95% ethanol extract using silica-gel column chromatography. The chemical structure of compound **1** was identified as AMF based on the ESI–MS, ^1^H- and ^13^C-NMR, and UV data (Table S1 and Figure S1). This is the first report on the isolation of AMF from VS. Furthermore, the HPLC profiles of VS extract were completely different from those of three other *Viburnum* species (*V. lantana* L., *V. opullus* var. sargentii (Koehne) Takeda, and *V. dillatatum* Thunb.) (Figure 1D–G). VS extract contained a high content of AMF (29.4 mg/g extract). The extracts of the three other *Viburnum* species did not contain AMF.

### 2.2. Effect of VS Extract on Body and Organ Weights

**Figure 2:**
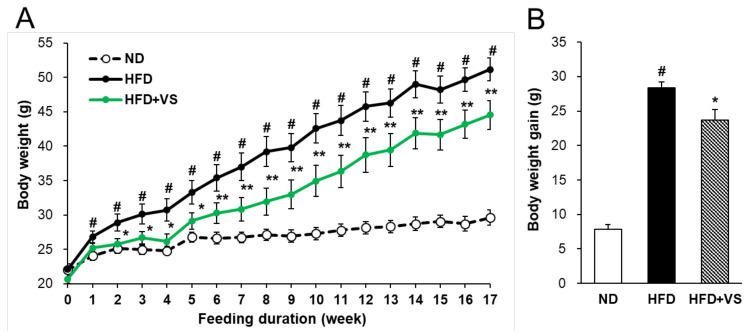
Effect of VS extract on body weight in high-fat diet (HFD)-fed mice. (**A**) Weekly monitored body weights and (**B**) 17-week cumulative body weight gains. Data are presented as means ± SE (*n* = 8). # indicates at *P* < 0.01 vs. the normal diet (ND) group; * indicates *P* < 0.05, ** indicates *P* < 0.01 vs. the HFD group.

To determine whether VS extract has an anti-obesity effect in HFD-fed mice, individual body weights were recorded weekly during the experimental period (Figure 2A). The initial body weights of all the groups were not different. After 17 weeks, the body weight in the HFD group was significantly increased compared with that in the normal diet (ND) group. However, the body weight gain in the HFD+VS group was markedly reduced by 16.5% compared to that in the HFD group (Figure 2B).

Supplementation with VS extract had no effect on the food and energy intake in HFD-fed mice during the experimental diet period (Table 1). The liver weight of the HFD group was 1.28-fold higher than that of the ND group. The weights of the liver decreased considerably (by 29.9%) in the HFD+VS group compared to the HFD group, whereas treatment with VS extract did not affect the weights of the pancreas or muscle. In addition, supplementation with VS extract considerably decreased the weights of the retroperitoneal, gonadal, and inguinal adipose tissue (AT) (6.4%, 17.9%, and 22.5%, respectively). Accordingly, the weight of total WAT also significantly decreased by 17.7% in the HFD+VS group compared to the HFD group. These data indicate that VS extract may exert anti-obesity effects by decreasing body weight gain and fat weight in vivo, without affecting the amount of food and energy intake.

### 2.3. Effect of VS Extract on Metabolic Parameters and Glucose Homeostasis

**Figure 3:**
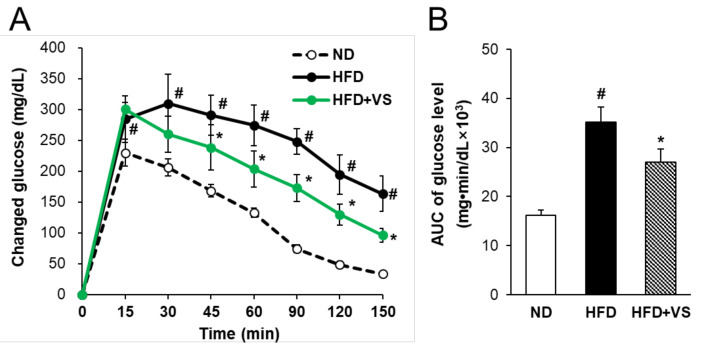
Effects of VS extract on oral glucose tolerance test (OGTT) results in HFD-fed mice. The OGTT was performed in the 14th week after the start of the oral gavage of 150 mg·kg^−^^1^·day^−^^1^ VS extract in HFD-fed mice. (**A**) Changes in blood glucose levels were assessed after glucose administration (2 g per kilogram of body weight). Blood glucose level was measured in tail vein blood at 0, 15, 30, 45, 60, 90, 120, and 150 min. (**B**) Area under the curve (AUC) of changed glucose level during OGTT. Data are presented as means ± SE (*n* = 8). # indicates *P* < 0.01 vs. the ND group; * indicates *P* < 0.05 vs. the HFD group.

After 14 weeks of feeding, an oral glucose tolerance test (OGTT) was performed to determine the effect of VS extract on the glucose tolerance in HFD-fed mice (Figure 3A). The glucose clearance ability of the HFD group was significantly lower than that of the ND group. The peak blood glucose concentration after glucose administration appeared at 30 min in the HFD group, while it was shifted to 15 min in the HFD+VS group. The blood glucose level of the HFD+VS group was significantly lower than that of the HFD group at 45, 60, 90, 120, and 150 min after oral glucose administration. Additionally, the area under the curve (AUC) for the integrated plasma glucose concentration was significantly reduced (by 23.0%) in the HFD+VS group compared to that in the HFD group (Figure 3B). These results indicate that VS extract effectively improved glucose tolerance in HFD-fed mice.

After 17 weeks of feeding, the plasma metabolic parameters of glucose, insulin, HOMA-IR, TG, and total cholesterol (TC) were significantly increased in the HFD group compared with those in the ND group (Table 2). However, glycated hemoglobin (HbA1c) and non-esterified fatty acid (NEFA) levels were almost comparable between all groups. The addition of VS extract markedly reduced glucose and insulin levels by 11.3% and 45.5%, respectively, in the HFD-fed mice. Accordingly, the HOMA-IR index was significantly decreased by 60.8% in the HFD+VS group compared to that in the HFD group. Plasma TG and TC levels were reduced by 17.2% and 8.9%, respectively, in the HFD+VS group. The plasma levels of aspartate aminotransferase (AST) and alanine aminotransferase (ALT), parameters of liver function, were significantly higher in the HFD group than those in the ND group, but the levels were still within the normal range. These markers were significantly lower in the HFD+VS group (23.0% and 48.7%, respectively) than those in the HFD group. These results suggest that VS extract may have moderating effects on hyperglycemia and hyperlipidemia.

### 2.4. Effect of VS Extract on Adipocyte Hypertrophy and Insulin Sensitivity in WAT

**Figure 4:**
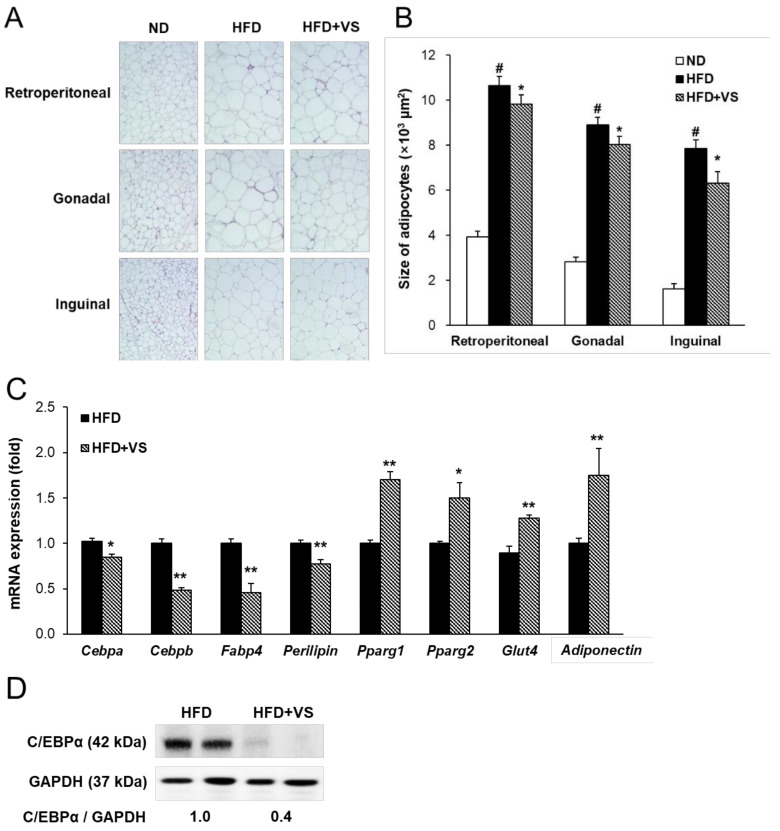
Effects of VS extract on the regulation of adipogenesis and insulin sensitivity in white adipose tissues (WATs) of HFD-fed mice. (**A**) Histology of WATs stained with hematoxylin and eosin (× 200 magnification). (**B**) Quantitative measurement of adipocyte sizes. (**C**) The levels of mRNA and (**D**) protein were measured by real-time qRT-PCR and Western blot, respectively, in the inguinal adipose tissue. The mRNA levels were normalized using glyceraldehyde-3-phosphate dehydrogenase (*Gapdh*) as a reference gene. Data are presented as means ± SE (*n* = 8). # indicates *P* < 0.01 vs. the ND group; * indicates *P* < 0.05, ** indicates *P* < 0.01 vs. the HFD group.

WAT, an insulin-sensitive tissue, plays a key role in regulating systemic energy homeostasis as fat storage. Hematoxylin and eosin (H&E) staining was performed to estimate the adipocyte size of the retroperitoneal, gonadal, and inguinal ATs. The average size of adipocytes in the HFD group was significantly higher than that in the ND group. Supplementation with VS extract markedly decreased the adipocyte size of the gonadal and inguinal AT by 9.9% and 19.5%, respectively, but not in the retroperitoneal AT (Figure 4A,B).

Furthermore, we examined the mRNA levels of adipogenesis-related genes to evaluate whether VS extract affected adipocyte differentiation in inguinal AT, which is sensitive to insulin as subcutaneous fat (Figure 4C). The mRNA levels of *Cebpa* and *Cebpb*, the key regulators of adipogenesis, were markedly decreased by treatment with VS extract. Supplementation with VS extract significantly reduced the mRNA levels of fatty acid binding protein 4 (*Fabp4*; known as *aP2* in adipose tissue) and *Perilipin*, which are late markers of adipocyte differentiation. Protein expression of C/EBPα also showed a considerable reduction in the HFD+VS group compared to that in the HFD group (Figure 4D). In addition, we observed that treatment with VS extract markedly enhanced the mRNA levels of insulin sensitivity-related genes, including *Pparg1*, *Pparg2*, and glucose transporter 4 (*Glut4*), compared to those in the HFD group (Figure 4C). Conversely, the mRNA level of *Adiponectin*, which is an adipocytokine and an important mediator of insulin action by adipose *Pparg* [13], was significantly increased in the HFD+VS group. These findings indicate that the anti-obesity and glucose-lowering effects of VS may be due to the inhibition of HFD-induced adipocyte hypertrophy by suppressing adipocyte differentiation, accompanied by enhanced insulin sensitivity.

### 2.5. Effect of VS Extract on HFD-induced Hepatic Steatosis

**Figure 5:**
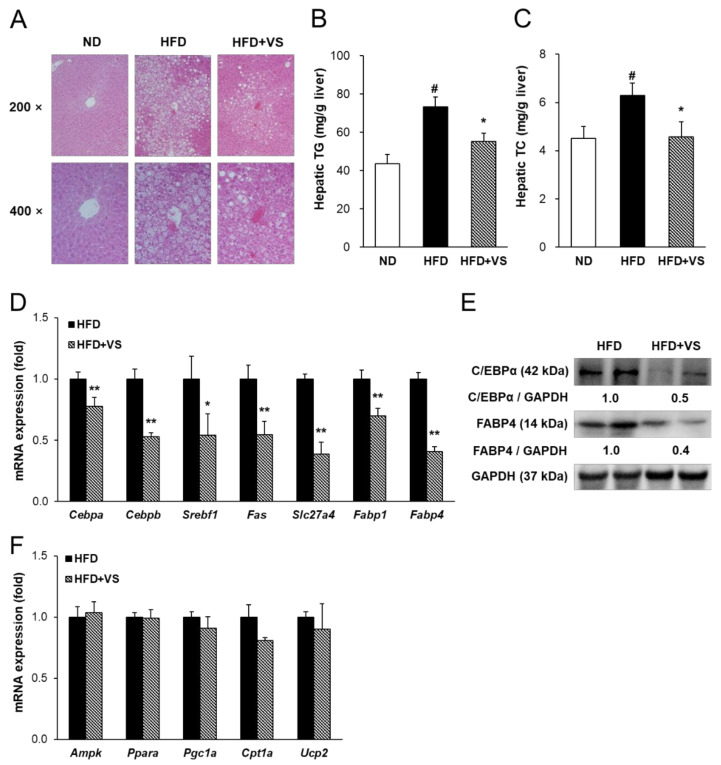
Effects of VS extract on the regulation of hepatic lipid metabolism in HFD-fed mice. (**A**) Histology of livers stained with hematoxylin & eosin (H&E) (× 200 and 400 magnification). (**B**) Hepatic triglycerides (TG) and (**C**) total cholesterol (TC) contents. (**D**,**F**) Relative hepatic mRNA levels were measured by real-time qRT-PCR and normalized using *Gapdh* as a reference gene. (**E**) Western blot of hepatic CCAAT/enhancer-binding protein α (C/EBPα) and fatty acid binding protein 4 (FABP4) detected with specific antibodies. Data are presented as means ± SE (*n* = 8). # indicates *P* < 0.01 vs. the ND group; * indicates *P* < 0.05, ** indicates *P* < 0.01 vs. the HFD group.

H&E staining of the liver tissue from each group is shown in Figure 5A. Hepatic steatosis was observed with the accumulation of many lipid droplets in the HFD group compared to that in the ND group. In contrast, supplementation with VS extract effectively suppressed HFD-induced hepatic lipid accumulation. Correspondingly, the hepatic TG and TC contents were significantly decreased by 24.8% and 27.0%, respectively, in the HFD+VS group compared to those in the HFD group (Figure 5B,C).

Next, transcription factors and target genes related to lipogenesis, fatty acid transport, and lipolysis in the liver were investigated in HFD-fed mice (Figure 5D,F). Consistent with the downregulated expression in the inguinal AT, the mRNA levels of *Cebpa* and *Cebpb* were significantly decreased in the livers of HFD-fed mice treated with VS extract. Supplementation with VS extract also markedly reduced the mRNA levels of sterol regulatory element-binding transcription factor 1 (*Srebf1*) and its target gene, fatty acid synthase (*Fas*). There was also a significant downregulation of the mRNA levels of genes related to free fatty acid transport in liver tissue, including solute carrier family 27 member 4 (*Slc27a4*), *Fabp1*, and *Fabp4* in the HFD+VS group. As shown in Figure 5E, VS extract markedly suppressed the hepatic protein expression of C/EBPα and FABP4 compared to that in the HFD group. However, administration of VS extract did not affect the mRNA levels of lipolysis-related factors, including AMP-activated protein kinase (*Ampk*), *Ppara*, peroxisome proliferator activated receptor gamma coactivator 1α (*Pgc1a*), carnitine palmitoyltransferase 1α (*Cpt1a*), and uncoupling protein 2 (*Ucp2*) (Figure 5F). These results suggest that VS extract improved HFD-induced hepatic steatosis by inhibiting lipid biosynthesis and transport.

### 2.6. Effects of VS Extract on Fatty Acid β-oxidation and Insulin Sensitivity in the Skeletal Muscle

**Figure 6:**
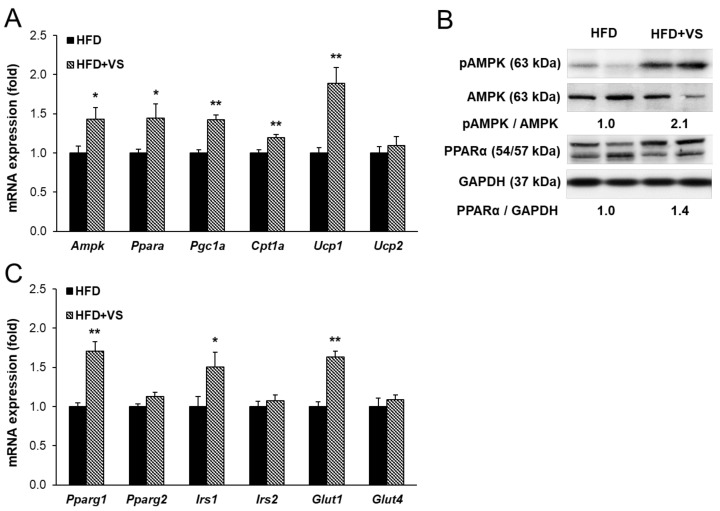
Effects of VS extract on the regulation of fatty acid β-oxidation and insulin sensitivity in the skeletal muscle of HFD-fed mice. (**A**),(**C**) Relative muscular mRNA levels were measured by real-time qRT-PCR and normalized using *Gapdh* as a reference gene. (**B**) Western blot of muscular PPARα, phospho- AMP-activated protein kinase (AMPK), and AMPK detected with specific antibodies. Data are presented as means ± SE (*n* = 8). * indicates *P* < 0.05, ** indicates *P* < 0.01 vs. the HFD group.

Skeletal muscle plays a key role in regulating whole-body energy consumption through insulin-stimulated glucose uptake and lipid oxidation [14]. Figure 6A shows that administration of VS extract significantly enhanced the fatty acid oxidation-related factors, including *Ampk*, *Ppara*, *Pgc1a*, and *Cpt1a*, in HFD-induced obese skeletal muscle. Additionally, we examined the mRNA levels of *Ucp1* and *Ucp2* involved in energy metabolism and thermogenesis in skeletal muscle. Supplementation with VS extract increased the mRNA expression of *Ucp1*, while there was no significant change in *Ucp2*. Moreover, the protein levels of PPARα and phosphorylated AMPK were markedly increased following treatment with VS extract (Figure 6B). In addition, we found a significant increase in the gene expression of *Pparg1* in the HFD+VS group compared to that in the HFD group, but not in that of *Pparg2* (Figure 6C). Supplementation with VS extract upregulated the mRNA levels of insulin signaling-related genes, including insulin receptor substrate 1 (*Irs1*) and *Glut1*, while the *Irs2* and *Glut4* levels were not changed. These results indicate that VS extract may improve muscular fatty acid β-oxidation and insulin sensitivity.

### 2.7. AMF Regulates Lipogenesis and Insulin Signaling in HFD-Fed Mice

**Figure 7:**
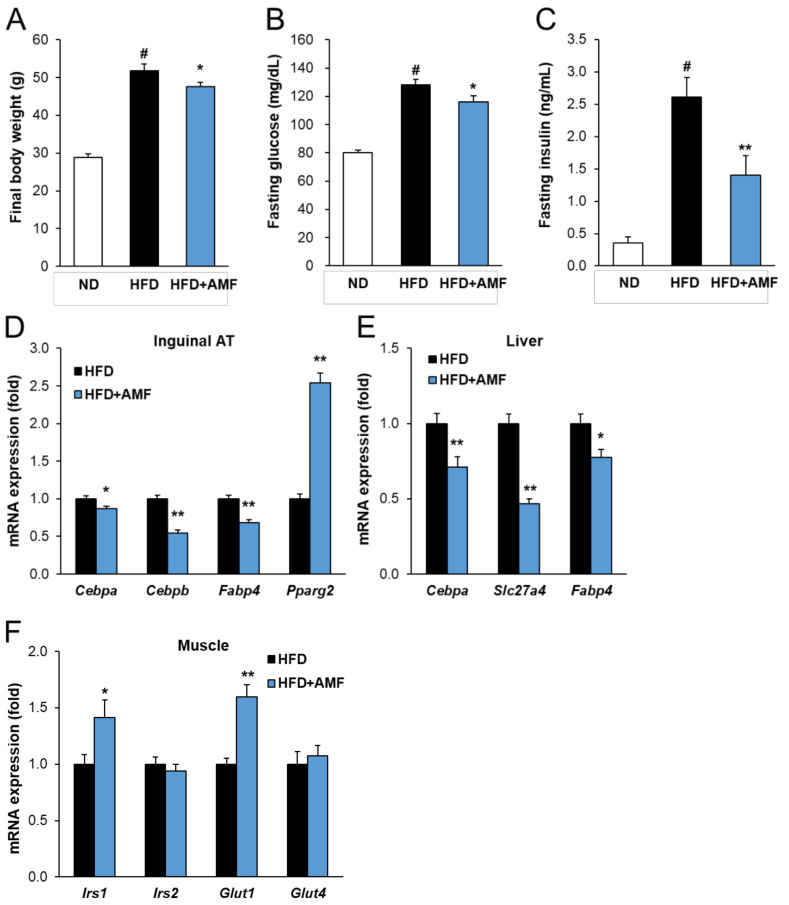
Effects of amentoflavone (AMF) in HFD-fed mice. (**A**) Body weights, (**B**) fasting glucose levels, and (**C**) fasting insulin levels were measured after 17 weeks. (**D**) Relative mRNA levels were measured by real-time qRT-PCR in the inguinal adipose tissue (AT), (**E**) liver, and (**F**) skeletal muscle and normalized using *Gapdh* as a reference gene. Data are presented as means ± SE (*n* = 8). # indicates *P* < 0.01 vs. the ND group; * indicates *P* < 0.05, ** indicates *P* < 0.01 vs. the HFD group.

To confirm the anti-obesity effect of AMF, which is enriched in VS extract, we performed Experiment 2 on mice fed the experimental diet supplemented with 10 mg/kg body weight of AMF. After 17 weeks of feeding, the final body weight of the HFD group was significantly higher than that of the ND group (Figure 7A). In contrast, HFD-fed mice supplemented with AMF gained significantly less weight compared to the HFD group. In addition, plasma levels of fasting glucose and insulin were also markedly reduced by 9.4% and 46.1%, respectively, after AMF treatment (Figure 7B,C). The addition of AMF significantly decreased the mRNA levels of the lipogenesis-related genes *Cebpa*, *Cebpb*, *Fabp4*, and *Slc27a4* in the inguinal AT or liver (Figure 7D,E). *Pparg2* expression in the HFD+AMF group was markedly increased in inguinal AT compared to that in the HFD group. Furthermore, the addition of AMF upregulated the mRNA levels of insulin signaling-related genes, including *Irs1* and *Glut1*, in the skeletal muscle, while the *Irs2* and *Glut4* levels were not changed (Figure 7F). These data confirmed that AMF may be the bioactive compound of VS extract on obesity and hyperglycemia by regulating lipogenesis and insulin signaling.

## 3. Discussion

Despite being a common species in Costa Rica and Panama, little is known about the components and bioactivities of VS, except for its taxonomic properties and dietary availability of its berries. However, several studies have reported the health benefits of congeneric plants of *Viburnum* in diabetic animals. The fruits of *V. dilatatum* Thunb., which is a wild deciduous low tree found widely in northern Japan, has been reported to decrease oxidative stress in organs, body weight, and hyperglycemia in streptozotocin-induced diabetic rats via the action of its key compound, cyanidin 3-sambubioside [15,16]. Recently, the phenolic-rich fraction comprised of procyanidin, catechin, and chlorogenic acid from the berries of *V. opulus* L. has been reported to potentially delay the rate of glucose and fatty acid absorption by intestinal cells and effectively decrease lipid accumulation in Caco-2 cells [17]. In the present study, we investigated whether VS extract exerts anti-obesity and anti-hyperglycemic effects in HFD-fed C57BL/6J mice. These mice have been used as an animal model for human obesity because HFD-induced obesity in this strain clearly expresses features common to human metabolic syndrome [18]. During the 17-week experimental period, the results of plasma analysis and OGTT showed that long-term HFD feeding caused hyperglycemia and glucose intolerance. VS extract effectively elicited anti-obesity activity with a significant decrease in plasma glucose, insulin, HOMA-IR, and TG levels. Excess lipid accumulation was demonstrated by the hepatic TG contents in the livers of HFD-fed mice, whereas VS extract considerably ameliorated fatty liver, thereby improving hepatic steatosis. In addition, supplementation with VS extract markedly decreased the mRNA levels of lipid metabolism-related genes, including *Cebpa*, *Cebpb*, *Fabp4*, and *Perilipin*, in inguinal AT, accompanied by a reduction of lipogenesis-related genes, including *Srebf1*, *Fas*, and *Slc27a4*, in the liver. Thus, our findings reveal for the first time that VS extract exerts anti-obesity effects by regulating hyperglycemia, lipogenesis, and lipid metabolism in HFD-fed mice.

In obesity and diabetes, adipose tissue expands by increasing the size of adipocytes (hypertrophy), which damages insulin sensitivity [1]. Adiponectin is highly expressed in WAT, and circulating adiponectin levels are diminished in obesity-related chronic disorders. Adiponectin inhibits hepatic gluconeogenesis and promotes fatty acid oxidation in skeletal muscle [7]. When the skeletal muscle is exposed to excessive lipids during obesity, fatty acids are not easily consumed via β-oxidation. As a result, fatty acid-derived metabolites accumulate inside the skeletal muscle. Moreover, skeletal muscle is a major site of insulin-stimulated glucose clearance. Obesity-related glucose intolerance may occur as a consequence of the metabolic overload of muscle mitochondria [19]. As observed in the present study, supplementation with VS extract increased the levels of fatty acid β-oxidation-related genes, including *Ampk*, *Ppara*, *Pgc1a*, and *Cpt1a*. AMPK is an enzyme that controls energy homeostasis and plays a key role in the metabolism of glucose and fatty acids. AMPK functions in various organs; however, AMPK was particularly activated in the skeletal muscle after treatment with VS extract in this study, and the activated AMPK might improve the uptake and oxidation of fatty acids and glucose transport [20]. Fatty acids are absorbed into the cell by the FABP family and are stored by the PPAR family, which are transcription factors activated by PGC1α. Phosphorylated AMPK enhances the activities of PPARα and its cofactor PGC1α, which are involved in mitochondrial biogenesis and function. The activation of long-chain fatty acids is catalyzed by a long-chain fatty acid coenzyme A ligase (FACL) to form acyl-CoA and enter the mitochondria through the CPT complex and is used as acetyl-CoA [4,8]. In addition, this study describes the mRNA profiles of the insulin signaling pathway in the inguinal AT and skeletal muscle of HFD-fed mice. Supplementation with VS extract was found to increase *Pparg* gene expression, which is known to enhance the insulin signaling pathway by improving glucose uptake [21]. Glucose enters the cell via GLUTs, regulated by PPARγ [13], and is used or stored in the form of pyruvate and glycogen. Supplementation with VS extract increased the mRNA level of *Glut4* in the inguinal AT. In contrast, treatment with VS extract activated insulin signaling by upregulating the mRNA expression of *Irs1* and *Glut1* in the skeletal muscle but did not affect *Irs2* and *Glut4* expression. GLUT1, which is primarily localized on the cell surface, is the major glucose transporter in the basal state of skeletal muscle. However, GLUT4 is expressed exclusively in skeletal muscle and fat cells, but the *Glut4* mRNA level is mostly unchanged in skeletal muscle of non-insulin-dependent diabetes mellitus [22,23]. Additionally, insulin-like signaling via IRS1/2 is essential for the regulation of skeletal muscle growth and metabolism for metabolic homeostasis. IRS1 contributes mainly to insulin secretion, whereas IRS2 is more critical for pancreatic β-cell growth, function, and development [24]. In insulin target tissues, including muscle and AT, IRS1 acts as a major substrate for the stimulation of glucose transport [25]. Insulin binding to the α-subunits of its receptor triggers the tyrosine kinase activity of the β-subunits, and the phosphorylated insulin receptor phosphorylates the tyrosine residues of IRS1. Insulin regulates glucose homeostasis by increasing glucose transport in skeletal muscle [26]. Therefore, VS extract enhanced fatty acid β-oxidation by increasing glucose uptake and improving insulin sensitivity.

Previous studies have reported that high concentrations of AMF were detected in the section Lantana Spach, especially in the 95% ethanol extract of *V. glomeratum* Maxim. leaves, which contained the highest concentration of AMF (5.46 mg/g extract) [10]. In comparison, the 95% ethanol extract of VS aerial parts contained much higher contents of AMF (29.4 mg/g extract) in this study. Furthermore, HPLC profiles of the VS extract showed that its main component was AMF, while those of the extracts from the aerial parts of the other *Viburnum* species (*V. lantana* L., *V. opullus* var. sargentii (Koehne) Takeda, and *V. dillatatum* Thunb.) were nearly not contained AMF. Moreover, the present study elucidated the in vivo molecular mechanism of AMF for the first time, indicating that it exerts anti-obesity and glucose-lowering effects by improving insulin sensitivity through upregulating the mRNA expression of *Irs1* and *Glut1*. These results suggest that the enriched AMF in VS extract may contribute to the suppression of obesity and hyperglycemia in HFD-fed mice.

There are some limitations to this study. Adipose tissue is a complex and active secretory organ that sends and receives signals that modulate energy expenditure, insulin sensitivity, inflammation, and immunity. WAT is infiltrated by macrophages with chronic low-grade inflammation in obesity. These infiltrating macrophages release pro-inflammatory cytokines, including TNFα, IL-6, and IL-1β, which are nuclear factor-kappa B (NF-κB) target genes [1,27]. Inflammatory mediators, including free fatty acids, IL-6, TNFα, and monocyte chemotactic protein-1, are spread through systemic circulation. They activate the c-Jun N-terminal kinases and NF-κB signaling pathways, promoted by the phosphorylation of IRS-1 at serine sites in the liver and skeletal muscle, resulting in the inhibition of insulin signaling [1,28]. Future studies should aim to clarify whether VS extract regulates obesity-induced low-grade inflammation via macrophage infiltration.

## 4. Materials and Methods

### 4.1. Preparation of VS and HPLC Analysis

The aerial parts of VS were harvested and dried in Costa Rica before being delivered from the International Biological Material Research Center (IBMRC) of the Korea Research Institute of Bioscience and Biotechnology (KRIBB, Daejeon, Korea). The dried aerial parts of VS (100 g) were pulverized, ground, and extracted with 95% ethanol (1 L) for 48 h at 25–30 °C. The whole extract was filtered and evaporated under reduced pressure at a temperature below 40 °C to obtain 95% ethanol extract of VS (8.7 g). The 95% ethanol extracts of three *Viburnum* species (*V. lantana* L., *V. opullus* var. sargentii (Koehne) Takeda, and *V. dillatatum* Thunb.) were obtained from the IBMRC of KRIBB.

The extracts of four *Viburnum* species were analyzed and confirmed using an HPLC system equipped with a diode-array detector system (Shimadzu Corporation, Tokyo, Japan) and a Brownlee SPP C18 column (4.6 mm × 50 mm, 2.7 μm; Perkin Elmer, Inc., Waltham, MA, USA). The injection volume was 5 μL, and the mobile phase was composed of 0.1% acetic acid in water (solvent A) and acetonitrile (solvent B). The linear gradient elution program was set to 5–30% B for 0–20 min, 30–100% B for 15–22.5 min, 100% B for 22.5–25 min, 100–5% B for 25–27.5 min, and 5% B for 27.5–30 min. The flow rate was 1.8 mL/min, and the absorbance of the HPLC profile was 254 nm. AMF was used as an external standard (Sigma-Aldrich, St. Louis, MO, USA).

### 4.2. Animals and Diets

The animal experimental protocols were approved by the Animal Care and Use Committee of KRIBB (KRIBB-ACE-18216) and performed in compliance with the Animal Research Reporting In Vivo Experiment (ARRIVE)-based guidelines of the KRIBB.

#### 4.2.1. Experiment 1

Four-week-old male C57BL/6J mice were purchased from the Laboratory Animal Resource Center of the KRIBB and kept under controlled temperature (22 ± 2 °C), humidity (50 ± 5%), and lighting (12 h light/dark cycle). They were provided free access to autoclaved water and gamma-irradiated diets in a specific pathogen-free facility in KRIBB. The mice were weaned onto a normal diet (3.82 kcal/g, 10% fat, 20% protein, 70% carbohydrate) (D12450B; Research Diet, Inc., New Brunswick, NJ, USA) for three weeks and then randomized into three separate treatment groups (*n* = 8/group): an ND group (supplemented with 10 kcal% fat); an HFD group, fed a 60 kcal% fat diet (5.21 kcal/g, 60% fat, 20% protein, 20% carbohydrate) (D12492; Research Diet, Inc.) with no supplementation; and an HFD+VS group, fed an HFD with oral administration of 150 mg/kg VS extract once a day for 17 weeks. VS extract was dissolved and diluted in sterile purified water at 15 mg/mL containing 10% polyethylene glycol and 0.5% Tween-80.

#### 4.2.2. Experiment 2

The effects of AMF supplementation for 17 weeks on obesity and hyperglycemia were measured under the same conditions as those in Experiment 1. Briefly, acclimated mice were divided into three groups (*n* = 8/group): an ND group, an HFD group with no supplementation, and an HFD+AMF group, which was fed an HFD with oral administration of 10 mg/kg AMF once a day for 17 weeks. AMF was dissolved in 0.1% dimethyl sulfoxide and diluted in sterile purified water at 1 mg/mL containing 10% polyethylene glycol and 0.5% Tween-80.

### 4.3. Measurements of Body and Organ Weights and Biochemical Parameters

Changes in body weight and food intake were monitored weekly. After 17 weeks of VS extract and AMF supplementation, the mice were fasted overnight. Fasting glucose levels were measured using an Accu-CheK Active glucometer (Roche, Mannheim, Germany). The mice were euthanized and the tissues were dissected out. Blood was collected into EDTA-coated tubes from the inferior vena cava of each mouse, and then centrifuged at 800× *g* for 15 min at 4 °C to obtain plasma and stored at −70 °C until analysis. HbA1c levels were estimated using an Easy A1c cartridge system (Infopia, Anyang, Korea). Insulin levels were measured using a Mouse Insulin Enzyme-Linked Immunosorbent Assay kit (Alpco Diagnostics, Salem, NH, USA). The concentrations of plasma TG, TC, AST, and ALT were assayed using available kits (Asan Pharm Co., Seoul, Korea). NEFAs were estimated using an automated blood chemistry analyzer (Hitachi-7150; Hitachi Medical, Tokyo, Japan). The HOMA-IR index was calculated using the following formula: HOMA-IR index = [fasting insulin concentration (ng/mL)] × 24.8 × [fasting glucose concentration (mg/dL)]/405 [29].

### 4.4. Oral Glucose Tolerance Test (OGTT)

After 14 weeks of feeding, an OGTT was performed. The mice were fasted for 16 h before the OGTT and orally administered glucose (2 g/kg body weight). Glucose levels were measured in blood collected from the tail vein at 0, 15, 30, 45, 60, 90, 120, and 150 min after glucose administration using a glucometer. The OGTT curves expressed the changed glucose levels. The AUC after the OGTT was calculated using the trapezoidal method.

### 4.5. Histological Analysis of WAT and Liver

To characterize the histological alterations, WAT and liver tissue from each mouse were dissected after sacrifice, fixed in 10% formaldehyde, and processed for paraffin embedding. Then, the samples were sliced into 4-µm-thick sections and stained with H&E. The morphology of the stained tissues was obtained using an Olympus BX61 microscope system equipped with an Olympus DP71 digital camera (Olympus, Tokyo, Japan). The size of the adipocytes was measured using the MetaMorph Imaging System (Meta Imaging Software, Sunnyvale, CA, USA).

### 4.6. Measurement of Hepatic Lipid Contents

Hepatic TG and TC were extracted from the liver using the Folch et al. method [30]. The concentrations were measured using the same enzymatic kits used for the plasma analyses.

### 4.7. Quantitative Real-Time RT-PCR (qRT-PCR)

The muscle, liver, and inguinal adipose tissue were anatomized and soaked in an RNAlater solution (Qiagen, Valencia, CA, USA) for RNA extraction. Total RNA was isolated from each tissue using TRIzol reagent (Ambion, Foster City, CA, USA), followed by additional purification with a RNeasy Mini Kit (Qiagen). Total RNA was used to synthesize cDNA using a High-Capacity cDNA Reverse Transcription kit (Thermo Fisher Scientific, Inc., Waltham, MA, USA). qRT-PCR was performed on a 7500 real-time PCR system (Applied Biosystems, Foster City, CA, USA) using specific primers that were designed with Primer-Express v3.0.0 software (Applied Biosystems). RNA expression was quantified by running all samples in duplicate using a FastStart Universal SYBR Green Master Mix (Roche), which was incorporated into dsDNA along with oligos synthesized to amplify each gene. The gene expression level is indicated as the number of PCR cycles at a point where the cDNA is amplified and its fluorescence reaches saturation. The results were normalized using glyceraldehyde-3-phosphate dehydrogenase (*Gapdh*) as a reference gene, such that the resulting value was finally calculated as a relative value. The primers used in these experiments are listed in Table S2.

### 4.8. Western Blot Analyses

Mouse-tissue protein extracts were subjected to immunoblot analysis with specific antibodies against phospho-AMPK, AMPK, PPARα, C/EBPα (Santa Cruz Biotechnology, Santa Cruz, CA, USA), FABP4 (Cell Signaling Technology, Danvers, MA, USA), and GAPDH (Bioss Antibodies, Boston, MA, USA) for the analysis of phosphorylated or total protein expression. GAPDH served as a loading control. Tissue proteins were fractionated by electrophoresis on 10% or 12% SDS-PAGE gels and transferred onto polyvinylidene fluoride membranes. The membranes were blotted with antibodies specific for each protein. Protein expression was determined using an Immobilon Western Chemiluminescent HRP Substrate (Merck Millipore, Burlington, MA, USA) and LAS-4000 Luminescent-Image Analyzer (Fuji Photo Film, Tokyo, Japan). The intensities of the detected bands were quantified using Fujifilm Image MultiGauge (Fuji Photo Film, Tokyo, Japan).

### 4.9. Statistical Analyses

The results are expressed as means ± standard error (SE). Statistical differences between groups were analyzed using one-way analysis of variance in JMP^®^ software (SAS Institute Inc., Cary, NC, USA) with Tukey’s test. mRNA expression was analyzed using Student’s *t*-test to identify significant differences between the two groups. Results with a *P*-value of less than 0.05 were considered significant.

## 5. Conclusions

**Figure 8:**
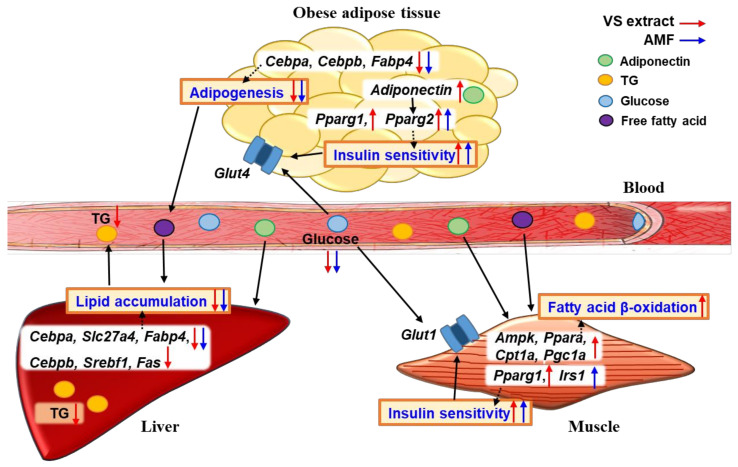
Possible molecular mechanisms of the anti-obesity and anti-hyperglycemic effects of VS extract and AMF. VS extract suppresses obesity and hyperglycemia by regulating C/EBPα-mediated lipogenesis, PPARα-mediated fatty acid β-oxidation, and PPARγ-mediated insulin sensitivity.

The present study reports the molecular mechanisms underlying the anti-obesity and glucose-lowering effects of VS extract by regulating C/EBPα-mediated lipogenesis, PPARα-mediated fatty acid β-oxidation, and PPARγ-mediated insulin sensitivity (Figure 8). Our findings indicate that AMF may be one of the active compounds of VS extract and suggest the usefulness of AMF-enriched VS extract as a natural anti-obesity agent with a glucose-lowering effect in the growth of C57BL/6J mice without any apparent toxicity or abnormalities.

## Figures and Tables

**Table 1:** Effects of VS extract on organ weights in HFD-fed mice.

Parameters	ND	HFD	HFD+VS
Food intake (g/day)	2.5 ± 0.0	2.5 ± 0.1	2.4 ± 0.2
Energy intake (kcal/day)	9.8 ± 0.1	13.2 ± 0.6 ^#^	12.5 ± 0.9
Liver (g/kg body weight)	29.5 ± 0.5	37.8 ± 3.3 ^#^	26.5 ± 1.7 *
Pancreas (g/kg body weight)	4.1 ± 0.2	3.9 ± 0.2	3.7 ± 0.2
Muscle (g/kg body weight)	10.9 ± 0.5	9.2 ± 0.9	9.2 ± 0.7
WAT (g/kg body weight)			
Retroperitoneal AT	3.0 ± 0.3	26.7 ± 0.4 ^#^	25.0 ± 0.6 *
Gonadal AT	13.8 ± 0.9	61.4 ± 3.0 ^#^	50.4 ± 3.6 *
Inguinal AT	8.2 ± 0.9	54.6 ± 2.2 ^#^	42.3 ± 3.8 **
Total WAT	25.0 ± 1.9	138.9 ± 7.5 ^#^	114.3 ± 4.7 *

Data are presented as means ± SE (*n* = 8). ^#^ indicates *P* < 0.01 vs. the ND group; * indicates *P* < 0.05, ** indicates *P* < 0.01 vs. the HFD group. WAT, white adipose tissue; AT, adipose tissue.

**Table 2:** Effects of VS extract on plasma parameters in HFD-fed mice.

Parameters	ND	HFD	HFD+VS
Glucose (mg/dL)	76.9 ± 3.6	124.3 ± 3.8 ^#^	110.2 ± 5.7 *
HbA1c (%)	4.8 ± 0.1	5.2 ± 0.3	5.3 ± 0.2
Insulin (ng/mL)	0.3 ± 0.1	2.2 ± 0.2 ^#^	1.2 ± 0.3 *
HOMA-IR	1.4 ± 0.2	20.4 ± 2.7 ^#^	8.0 ± 1.8 **
TG (mg/dL)	146.6 ± 4.1	155.6 ± 5.9 ^#^	128.9 ± 8.4 *
TC (mg/dL)	92.9 ± 4.9	225.6 ± 8.7 ^#^	205.5 ± 3.4 *
NEFA (mEq/L)	2.1 ± 0.2	2.2 ± 0.2	2.1 ± 0.2
AST (IU/L)	47.9 ± 2.4	60.8 ± 2.8 ^#^	46.8 ± 4.7 *
ALT (IU/L)	12.1 ± 0.7	35.9 ± 3.7 ^#^	18.4 ± 3.2 *

Data are presented as means ± SE (*n* = 8). ^#^ indicates *P* < 0.01 vs. the ND group; * indicates *P* < 0.05, ** indicates *P* < 0.01 vs. the HFD group. ALT, alanine aminotransferase; AST, aspartate aminotransferase; HbA1c, glycated hemoglobin; HOMA-IR, homeostasis model assessment of insulin resistance; NEFA, non-esterified fatty acid; TC, total cholesterol, TG, triglycerides.

## Data Availability

Data are contained within the article or supplementary material.

## Supplementary Materials

The following supplementary figures and tables are available online. Table S1 and Figure S1: Isolation and chemical structure elucidation of amentoflavone (AMF) from *Viburnum stellato-tomentosum* (VS) extract. Table S2 Sequences of primers used for real-time qRT-PCR [31,32,33].

## Sample Availability

Samples of *Viburnum stellato-tomentosum* are available from the authors.

## Abbreviations

AMF	amentoflavone
AMPK	AMP-activated protein kinase
C/EBP	CCAAT/enhancer-binding protein
CPT1α	carnitine palmitoyltransferase 1α
FABP	fatty acid binding protein
FAS	fatty acid synthase
GAPDH	glyceraldehyde-3-phosphate dehydrogenase
GLUT	glucose transporter
HbA1c	glycated hemoglobin
HOMA-IR	homeostasis model assessment of insulin resistance
IR	insulin resistance
IRS	insulin receptor substrate
NEFAs	non-esterified fatty acids
OGTT	oral glucose tolerance test
PGC1α	peroxisome proliferator activated receptor gamma coactivator 1α
PPAR	peroxisome proliferator-activated receptor
SLC27A4	solute carrier family 27 member 4
SREBF1	sterol regulatory element-biding transcription factor 1
T2DM	type 2 diabetes mellitus
TNFα	tumor necrosis factor α
UCP	uncoupling protein
VS	*Viburnum stellato-tomentosum*
WAT	white adipose tissue
