# Supplementary material for: Viburnum stellato-tomentosum Extract Suppresses Obesity and Hyperglycemia through Regulation of Lipid Metabolism in High-Fat Diet-Fed Mice
Source: Molecules. 2021 Feb 17;26(4):1052. doi: 10.3390/molecules26041052 (PMC7922011; doi:10.3390/molecules26041052)
Supplement: Supplementary file 1 [file molecules-26-01052-s001.pdf]

Supplementary Materials

Viburnum stellato-tomentosum Extract Suppresses Obesity and Hyperglycemia through Regulation of Lipid Metabolism in High-Fat Diet-Fed Mice

Seona Cho [1,2], Hwa Lee [1], Jisu Han [1], Haneul Lee [1], Rosales Ovares Kattia [3], Zamora Villalobos Nelson [3], Sangho Choi [4], Soo-Yong Kim [4], Ho-Yong Park [1], Hye Gwang Jeong [2,*], and Tae-Sook Jeong [1,*]

[1] Industrial Bio-materials Research Center, Korea Research Institute of Bioscience and Biotechnology (KRIBB), Daejeon 34141, Republic of Korea
[2] College of Pharmacy, Chungnam National University, Daejeon 34134, Republic of Korea
[3] Instituto Nacional de Biodiversidad (INBio), Santo Domingo, Heredia, P. O. Box 22-3100, Costa Rica
[4] International Biological Material Research Center, KRIBB, Daejeon 34141, Republic of Korea

*Corresponding authors:
Tae-Sook Jeong – *Industrial Bio-materials Research Center, Korea Research Institute of Bioscience and Biotechnology (KRIBB), 125 Gwahak-ro, Yuseong, Daejeon 34141, Republic of Korea*; Phone: +82-42-860-4558; E-mail: tsjeong@kribb.re.kr; Fax: +82-42-861-2675
Hye Gwang Jeong – *College of Pharmacy, Chungnam National University, , 99 Daehak-ro, Yuseong, Daejeon 34134, Republic of Korea*; Phone: +82-42-821-5936; E-mail: hgjeong@cnu.ac.kr; Fax: +82-42-823-6566

1. Isolation and Identification of Amentoflavone (AMF)

Grounded aerial parts of *Viburnum stellato-tomentosum* (5 g) were extracted with 50 mL EtOH for 48 h at 25-30°C, and the extract was concentrated to yield a residue (437 mg). The residue was suspended in H_2O and partitioned with *n*-hexane (30 mL × 3), chloroform (30 mL × 3), ethyl acetate (EtOAc) (30 mL × 2), and butanol (40 mL), successively. The EtOAc layer (44.6 mg) was subjected to silica-gel column chromatography (18 × 110 mm) with 100% dichloromethane (DCM), DCM/MeOH, and MeOH to obtain compound **1** (15.8 mg).

Isolated compound **1** was identified using spectroscopic data (Table S1 and Figure S1) and HPLC-DAD monitoring with authentic standard compound of AMF (Text Figure 1), and comparison with previously published data [31-33]. ESI-MS was obtained on an Agilent 6410 triple quadrupole LC/MS system (Agilent Technologies, Palo Alto, CA, USA). NMR spectra were recorded on a JEOL JNM-ECA600 600MHz FT-NMR spectrometer (JEOL, Tokyo, Japan) and chemical shifts are expressed in δ values. The standard compound of AMF was obtained from Sigma-Aldrich (Seoul, Korea). This is the first report on the isolation of AMF from *V. stellato-tomentosum*.

Table S1. ESI-MS and NMR data for compound **1**.

ESI-MS (Positive ion): m/z 539 $[M+H]^+$ (Molar mass: 538.45 g mol^{-1})

^{1}H-NMR data: (CD_3OD) d 6.18 (1H, br s, H-6), 6.38 (1H, s, H-6″), 6.40 (1H, br s, H-8), 6.59 (1H, s, H-3″), 6.60 (1H, s, H-3), 6.72 (2H, d, J = 8.0 Hz, H-5‴, H-3‴), 7.12 (1H, dd, J = 8.0, 1.5 Hz, H-6′), 7.54 (2H, d, J = 8.0 Hz, H-2‴, H-6‴), 7.89 (1H, d, J = 8.0 Hz, H-5′), 7.95 (1H, d, J = 1.5 Hz, H-2′)

^{13}C-NMR data: (CD_3OD) d 166.0 (C-2), 102.3 (C-3), 184.6 (C-4), 163.4 (C-5), 98.4 (C-6), 166.4 (C-7), 93.5 (C-8), 159.8 (C-9), 105. 6 (C-10), 123.5 (C-1′), 131.0 (C-2′), 122.0 (C-3′), 161.6 (C-4′), 127.9 (C-5′), 116.6 (C-6′), 166.6 (C-2″), 101.8 (C-3″), 185.0 (C-4″), 163.8 (C-5″),

98.6 (C-6″), 162.8 (C-7″), 106.0 (C-8″), 159.6 (C-9″), 105. 3 (C-10″), 123.5 (C-1‴), 128. 2(C-2‴, C-6‴), 115.4 (C-3‴, C-5‴), 163.0 (C-4‴)

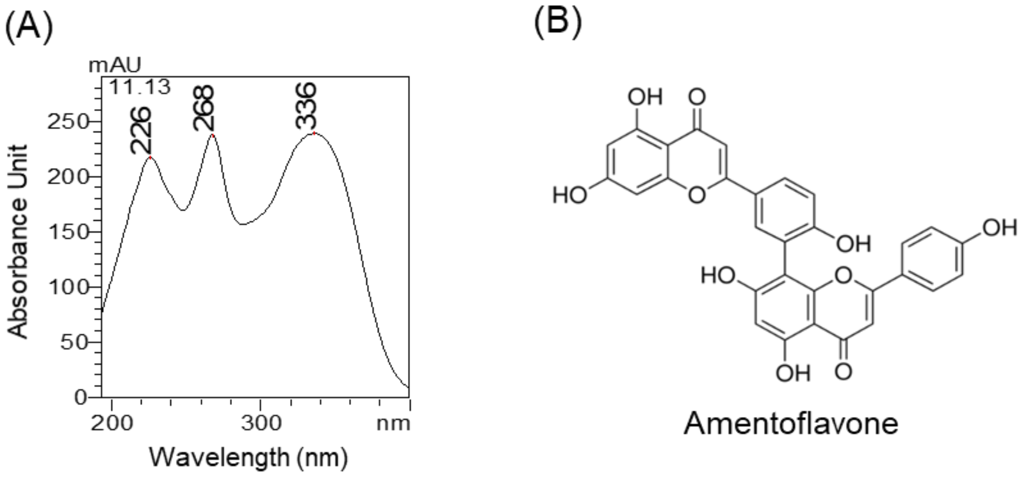

Figure S1. UV spectrum (A) and chemical structure (B) of compound **1**, amentoflavone.

Table S2. Sequences of primers used for real-time qRT-PCR

Gene (Number)	Forward Primer	Reverse Primer
Adiponectin (NM_009605.4)	CATGCCGAAGATGACGTTAC	CGATACACATAAGCGGCTTC
Ampk (NM_001013367.3)	TGGCTGAGAAGCAGAAGCAC	GGCCTGTCAATTGGTGTTCT
Cebpa (NM_007678)	CAAGAAGTCGGTGGACAAGA	TCAACTCCAGCACCTTCTGT
Cebpb (NM_009883)	AAGCTGAGCGACGAGTACAA	AGCTGCTTGAACAAGTTCCG
Cpt1a (NM_013495)	CTGCACTCCTGGAAGAAGAA	GTTCTTCGTCTGGCTTGACA
Fabp1 (NM_017399.5)	GCAAGTACCAATTGCAGAGCCAGG	TCATTGCGGACCACTTTGGG
Fabp4 (NM_024406)	TTTGTGGGAACCTGGAAGCT	CACGCCCAGTTTGAAGGAAA
Fas (NM_007988)	TGTGAGTGGTTCAGAGGCAT	TTCTGTAGTGCCAGCAAGCT
Gapdh (NM_001001303)	ACATCATCCCTGCATCCACT	AGATCCACGACGGACACATT
Glut1 (NM_011400.3)	AGCCGGCACAGCTAGAGCTT	TAGTCCGAGCACTGCTCCTC
Glut4 (NM_009204.2)	GCCCCACAGAAGGTGATTGA	AGCGTAGTGAGGGTGCCTTGT
Irs1 (NM_008386.3)	ACCCACCCAGGCTTTTGTC	CGGGACTTGGGTGTGTAGAAG
Irs2 (NM_001185083.1)	CCCCACCCAGGCTTTTGT	GCGGGACATGGGTGTGTAG
Perilipin (NM_175640.2)	TGGTACACACCGTGCAGAACA	TGGGAAGCGGCACATAGTG
Pgc1a (NM_008904)	GTGCAGCCAAGACTCTGTAT	GGTCGCTACACCACTTCAAT
Ppara (NM_011144)	CCTGAACATCGAGTGTCGAA	GTACTGGCATTTGTTCCGGT
Pparg1 (NM_001127330.2)	ATGTCTCACAATGCCATCAGGTT	GCGGGAAGGACTTTATGTATGAGT
Pparg2 (NM_011146.3)	TGTCTCACAATGCCATCAGGTT	AGCGGGAAGGACTTTATGTATGAGT
Slc27a4 (NM_011989.5)	GCCCTGCGCCACTGTCTTGA	TGGGCTCCAGGCTAGCATGG
Srebf1 (NM_011480)	GAGCGAGCGTTGAACTGTAT	ATGCTGGAGCTGACAGAGAA
Ucp1 (NM_009463)	CCAGGCTTCCAGTACCATTA	GCCACACCTCCAGTCATTAA
Ucp2 (NM_011671)	GCCTCTACGACTCTGTCAAA	CTTCGACAGTGCTCTGGTAT

References

31. Okigawa, M.; Hwa, C.W.; Kawano, N.; Rahman, W. Biflavones in *Selaginella* species. *Phytochemistry* **1971**, *10*, 3286–3287.
32. Lin, L.-C.; Kuo, Y.-C.; Chou, C.-J. Cytotoxic biflavonoids from *Selaginella delicatula*. *J. Nat. Prod.* **2000**, *63*, 627–630.
33. Ma, S.-C.; But, P.P.-H.; Ooi, V.E.-C.; He, Y.-H.; Lee, S.H.-S.; Lee, S.-F.; Lin, R.-C. Antiviral amentoflavone from *Selaginella sinensis*. *Biol. Pharm. Bull.* **2001**, *24*, 311–313.
